# RepSeq – A database of amino acid repeats present in lower eukaryotic pathogens

**DOI:** 10.1186/1471-2105-8-122

**Published:** 2007-04-11

**Authors:** Daniel P Depledge, Ryan PJ Lower, Deborah F Smith

**Affiliations:** 1 Immunology and Infection Unit, Department of Biology, University of York, Heslington, YORK, YO10 5YW, UK

## Abstract

**Background:**

Amino acid repeat-containing proteins have a broad range of functions and their identification is of relevance to many experimental biologists. In human-infective protozoan parasites (such as the Kinetoplastid and *Plasmodium *species), they are implicated in immune evasion and have been shown to influence virulence and pathogenicity. RepSeq  is a new database of amino acid repeat-containing proteins found in lower eukaryotic pathogens. The RepSeq database is accessed via a web-based application which also provides links to related online tools and databases for further analyses.

**Results:**

The RepSeq algorithm typically identifies more than 98% of repeat-containing proteins and is capable of identifying both perfect and mismatch repeats. The proportion of proteins that contain repeat elements varies greatly between different families and even species (3–35% of the total protein content). The most common motif type is the Sequence Repeat Region (SRR) – a repeated motif containing multiple different amino acid types. Proteins containing Single Amino Acid Repeats (SAARs) and Di-Peptide Repeats (DPRs) typically account for 0.5–1.0% of the total protein number. Notable exceptions are *P. falciparum *and *D. discoideum*, in which 33.67% and 34.28% respectively of the predicted proteomes consist of repeat-containing proteins. These numbers are due to large insertions of low complexity single and multi-codon repeat regions.

**Conclusion:**

The RepSeq database provides a repository for repeat-containing proteins found in parasitic protozoa. The database allows for both individual and cross-species proteome analyses and also allows users to upload sequences of interest for analysis by the RepSeq algorithm. Identification of repeat-containing proteins provides researchers with a defined subset of proteins which can be analysed by expression profiling and functional characterisation, thereby facilitating study of pathogenicity and virulence factors in the parasitic protozoa. While primarily designed for kinetoplastid work, the RepSeq algorithm and database retain full functionality when used to analyse other species.

## Background

All characterised eukaryotic proteomes contain proteins possessing repeated amino acid motifs within their sequence [1]. These repeats can arise from simple sequence repeats (termed SSRs) occuring in the coding regions of genomes. SSRs typically originate from unequal crossing-over and replication errors which result from the formation of unusual DNA structures such as slipped strands and hairpins [2,3]. SSRs range from single nucleotide repeats to large multi-codon repeats and are substantially more numerous in non-coding regions of the genome [4,5]. SSRs are also considered a major source of quantitative genetic variation [5-7].

A range of functions have been ascribed to amino acid repeats, the most common being that repeats are a mechanism for providing regular arrays of spatial and functional groups [8]. Error-prone SSR expansion allows for rapid evolution of proteins with repetitive structure, which can lead to rapidly changing phenotypes. In *Saccharomyces cerevisiae*, amino acid reiterations of different types are concentrated in different classes of proteins, including transcription factors, protein kinases and membrane transporters [9].

Proteins containing repeats are particularly widespread within several parasitic taxa including protozoan organisms that are the causative agents of malaria (*Plasmodium *species; [10]) and kinetoplastid parasites (*Trypanosoma *and *Leishmania *species that cause a range of debilitating human diseases (African Sleeping Sickness, Chagas' Disease and the Leishmaniases [11]). Known functions of intra-protein repeats include roles in intracellular protein-protein interactions, binding to host-cell receptors and polymerisation of their associated, non-repeated domains [12,13]. Protein repeats are also implicated in antigenic recognition and evasion of the host immune response to infection [14].

Proteins containing amino acid repeats are split into distinct categories based on different types of motif (Table 1).

**Table 1:** Examples of amino acid repeats.

Single Amino Acid Repeat (SAAR)	MJRK***EEEEEEEEEE***LKGT
Di-peptide Repeat (DPR)	MJRK***EDEDEDEDED***LKGT
Sequence Repeat Region (SRR)	MJRK***EEDKEEDKEEDK***GT

Each repeat type is shown within a sequence (bold, highlighted).

• Single amino acid repeats (SAARs) are uninterrupted runs of a single amino acid. Certain amino acids are more prevalent (usually alanine and glutamine), although this feature is usually species-specific [1].

• Di-peptide Repeat (DPRs) motifs occur when a pair of non-identical amino acids are tandemly repeated in a linear sequence. These are often referred to as tandem repeats.

• Sequence Repeat Regions (SRRs) occur when a given amino acid motif is repeated several times throughout a protein sequence. The length, number of repeats and amino acid content of the motif varies and can also include elements of SAARs and DPRs.

Consideration must also be given to so-called 'mismatch' repeats. These are characterised by repeat sequences in which mutations (insertions, deletions and substitutions) have rendered the sequences non-identical. While these may not affect the function of the repeat region, they do present an increased challenge for their identification. It has also been shown that mismatch repeats can be functionally important [15].

It is important to distinguish between repeats at the DNA level and repeats at the proteome level as only a small fraction of the repeats found in nucleotide sequences are translated into individual proteins. It is therefore much faster and simpler to analyse amino acid repeats at the protein level as search algorithms require less complexity and search a smaller amount of data.

While a large number of applications have been developed for detecting and analysing genome-level repeats (e.g. Repeat Masker [16], Repeat Scout [17] and Tandem Repeats Finder [18]), there are very few applications/databases which deal with proteome-level repeats. The available databases, including COPASAAR [1], ProtRepeatsDB [19] and TRIPS [20], mostly focus on prokaryotic and higher eukaryotic analyses. While COPASAAR and TRIPS deal with specific repeat-types (SAARs and tandemly repeated sequences, respectively), ProtRepeatsDB attempts to aggregate all repeat types into its database. Unfortunately the scope of the database and the complexity of the user interface are not suited to experimental biologists looking for less complex methods of quickly identifying repeat-containing proteins for *in vitro/in vivo *studies.

Of current importance is the creation of a database of repeats that clearly differentiates between the different repeat-types and provides enough options for both "quick and dirty" proteome analyses as well as more comprehensive proteome studies (such as inter-species analyses). This paper presents RepSeq, an online database of amino acid repeats found in lower eukaryotic pathogens.

## Construction and content

RepSeq (Database of Repeat Sequences) is a web-based database/application which allows the identification of all amino acid repeats within a given proteome. While primarily designed to work with lower eukaryotic pathogens, RepSeq can be used to study proteomes from any given organism. The RepSeq website houses an interactive database, an upload facility which uses the RepSeq algorithm to analyse user-provided sequences and all relevant documentation, methodology and glossary pages. RepSeq was devised by D. Depledge and implemented by R.J. Lower and D. Depledge.

### RepSeq algorithm

The RepSeq algorithm was written using PERL and identifies both perfect and mismatch repeats by searching for small regions with perfect identity within a protein sequence. The algorithm takes a FASTA formatted proteome file and uses a sliding window (6 residues in length) to search for repeated sequences. Each protein within the proteome is examined individually and all information collected is stored in conjunction with any information included in the sequence header (protein id, accession number, function etc). These data are then parsed to the RepSeq database. The algorithm functions by counting every 6-residue amino acid motif (termed a 'chunk') that appears within the protein sequence. The number of times each motif is repeated is recorded, together with its position within the protein sequence. The amino acid sequence of each chunk is also examined and a note made when a chunk contains a sequence of identical amino acids (i.e AAAAAA) or a 2-residue tandem repeat (i.e. ARARAR) In the case of SAARs and DPRs, the algorithm then deduces which chunks are part of the same repeat (based on the location and sequence of the chunks). For example, three identical chunks (i.e. AAAAAA) lying adjacent to each other will be identified as a SAAR, 8 residues in length.

All repeated chunks not classified as SAARs or DPRs are counted as SRRs and stored as such in the database. In addition, each SRR is given a score, based on the number of repeats of the chunks and their relative positions. This score can be used as an indicator of how strong a repeat motif is (i.e. a higher score indicates a strongly conserved motif repeated many times, whereas a low score indicates a less conserved motif repeated only a few times). The algorithm sensitivity is increased by including a function that allows for similar chunks (i.e. those in which 5 out of 6 residues are conserved) to be grouped together. This increases the chances of identifying lower identity repeat sequences but can also increase the number of false positives identified. Currently this is only implemented when identifying SRRs due to huge numbers of false positive DPRs and SAARs being identified. The RepSeq algorithm requires approximately 2–3 minutes to analyse a proteome of 10000 proteins on a P4 2.80GHz, 512MB RAM running Windows XP (SP2).

### RepSeq Testing

The RepSeq algorithm was evaluated using a number of test data sets, created to resemble lower eukaryotic proteomes in terms of size and proportion of repeat-containing proteins. The data sets generated comprised 5000 and 10000, of which 5% or 25% were repeat-containing proteins (containing SRR repeats of varying length and repetitions). The results are shown in Table 2 and discussed below. The test data sets were generated using a small script written in RUBY.

**Table 2:** Test data set analysis.

		**2+ SRRs**			**3+ SRRs**		
		
		**Loose Repeat Threshold**			**Loose Repeat Threshold**		
**Total Proteins**	**SRR Proteins**	Total	True positives	False positives	Total	True positives	False positives

**5000**	**250**	342	250 (100%)	92	248	248 (99.2%)	0
**5000**	**1250**	1306	1250 (100%)	56	1237	1237 (99.0%)	0
**10000**	**500**	674	500 (100%)	174	492	492 (98.4%)	0
**10000**	**2500**	2633	2500 (100%)	133	2466	2466 (98.6%)	0

							
		**Normal Repeat Threshold**			**Normal Repeat Threshold**		

**Total Proteins**	**SRR Proteins**	Total	True positives	False positives	Total	True positives	False positives

**5000**	**250**	256	250 (100%)	6	248	248 (99.2%)	0
**5000**	**1250**	1253	1248 (99.8%)	5	1237	1237 (99.0%)	0
**10000**	**500**	506	499 (99.8 %)	7	492	492 (98.4%)	0
**10000**	**2500**	2504	2496 (99.8%)	8	2466	2466 (98.6%)	0

							
		**Strict Repeat Threshold**			**Strict Repeat Threshold**		

**Total Proteins**	**SRR Proteins**	Total	True positives	False positives	Total	True positives	False positives

**5000**	**250**	245	245 (98.0%)	0	244	244 (97.6%)	0
**5000**	**1250**	1220	1220 (97.6%)	0	1219	1219 (97.5%)	0
**10000**	**500**	485	485 (97.0%)	0	484	484 (96.8%)	0
**10000**	**2500**	2424	2424 (97.0%)	0	2420	2420 (96.8%)	0

Proteomes containing 5000 or 10000 proteins (5% or 25% of which contained repeat regions) were created and analysed using RepSeq.

### RepSeq database

**Figure 1:**
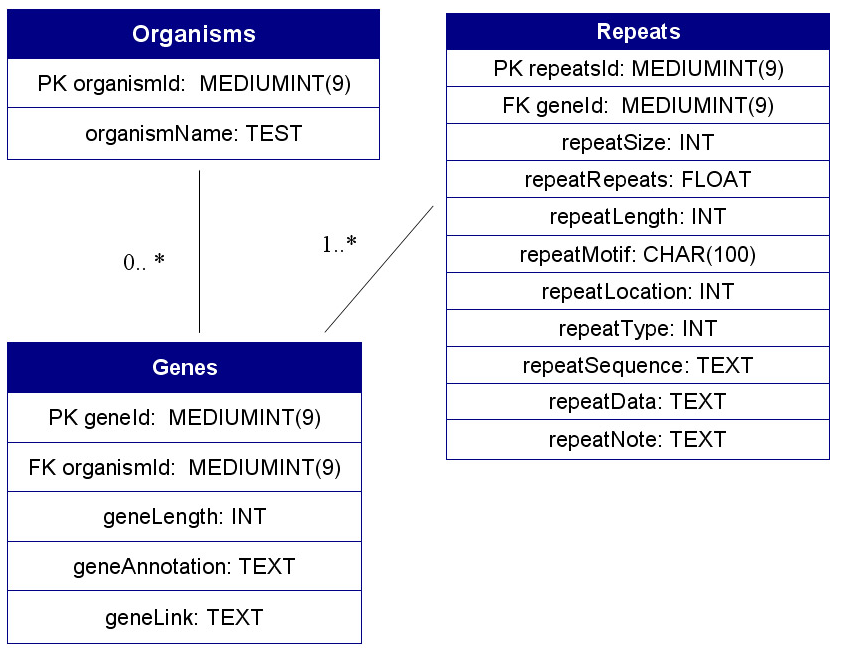
**RepSeq database UML design**. The database schema consists of three tables in which data redundancy is eliminated by data linking from child tables via foreign keys.

The database was written using MySQL v5.0 and consists of 3 tables, the schema and structure of which are shown in Figure 1. Given that each organism could house many repeat-containing proteins and that each gene could contain several distinct repeat-regions, it was necessary to structure the database so as to reduce data redundancy. Each table contains a unique and auto-incrementing column (*organismID, geneID and repeatID*) which allows data linking from child tables via foreign keys.

### User interface

**Figure 2:**
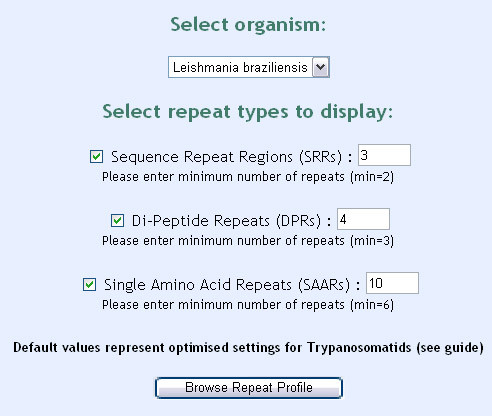
**RepSeq query interface**. The query interface contains a number of options that can be adjusted to limit/expand the search. The user is also able to search for specific genes or annotations.

**Figure 3:**
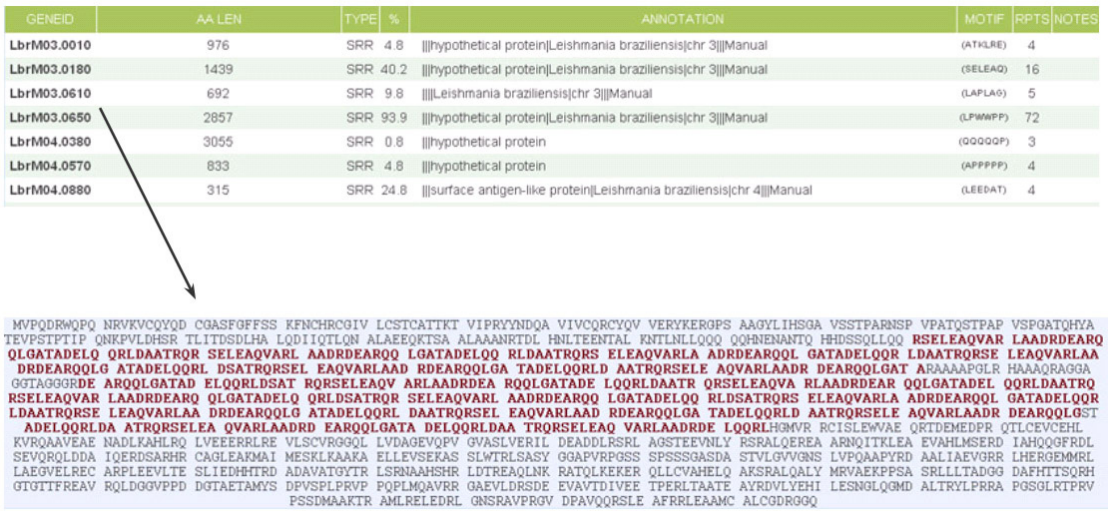
**RepSeq output table**. The top table shows the initial output of the input queries. Selecting a gene then displays the second image, indicating where each repeat is located (red) and allowing the user to determine its motif.

Access to the database is via the RepSeq web-interface (Figure 2). A variety of search criteria are present for examining the database. Users are able to specify which types of repeat they wish to search for, as well as assign values for the minimum length or number of repeat units. A repeat strength function also allows users to choose between loose, standard and strict searches. This function affects the number of repeat-containing proteins that are returned. Strict searches will only return proteins which possess strong repeats (based on the scoring system described previously) while loose searches will return all proteins containing any form of repeat. The relative strengths of these options are discussed below. Users are also able to search for specific proteins by their accession number or functional description. Once all options are specified, the data are retrieved from the database and displayed in an output table (Figure 3). Selecting proteins from the table will show the full protein sequence and highlight the repeated regions, allowing users to determine the full repeat motif. This method is faster and more reliable than any computational method so far developed.

### Upload facility

Proteomes from species not hosted at this site can be uploaded and analysed using the website's upload facility. Here, FASTA formatted files can be submitted for analysis. Results will be stored in the database for a minimum of seven days and a personalised link to a query page (specific to the uploaded proteome) will be provided. The dataset will also be available for download. The upload and processing time varies from 1–5 minutes for proteomes of 20000 proteins.

### RepSeq proteome data

The current database contains thirteen proteomes (Table 3). These were obtained from a variety of sources including GeneDB [21] and PlasmoDB [22] and are available for analysis using RepSeq. While primarily designed to aid the functional analysis of parasitic proteomes, the RepSeq database has been expanded to include a range of lower eukaryotic pathogens. Further proteomes will be added as they are sequenced and updated proteome releases will be incorporated as they become available.

**Table 3:** Protozoan parasite species currently available in RepSeq.

**Species**	**Predicted Protein-Coding Genes**
***L.braziliensis***	7046
***L.infantum***	8183
***L.major***	8302
***T.brucei***	8758
***T.cruzi***	25401
***T.congolense***	17203
***D.discoideum***	13498
***E.histolytica***	9766
***P.berghei***	12235
***P.chabaudi***	15007
***P.falciparum***	5479
***P.vivax***	5352
***P.yoelii***	8761

## Utility and discussion

Analysis of the test data sets (Table 2) indicates that the RepSeq algorithm functions properly and is able to identify all major repeat types. SAAR and DPR sequences are identified 100% of the time providing that they are of 6 residues or longer. In all test data sets, RepSeq identified 100% of SRRs when set to identify 2+ repeats on the 'loose' repeat strength threshold setting. This was counterbalanced by the identification of a large number of false positives. Increasing the SRR repeats to 3+, all false positives were removed while over 99.8% of repeat-containing proteins were identified. The "standard" setting (searching for 2+ repeats) identified 99.8% of repeat-containing proteins and registered a significantly smaller proportion of false positives (typically less than 10 in total). The "strict" setting reduced the proportion of repeat-containing proteins identified to 97% but did not detect any false positives. As mentioned earlier, RepSeq will also identify mismatch repeats (although allowing for 1 amino acid substitution) provided that two identical 6-residue sequences are conserved in the repeat. All the false positives identified are proteins containing one repeat of a 5/6 residue sequence and thus can easily be identified and removed from further analysis.

When analysing proteomes, consideration must be given to the fact that in any given amino acid sequence, a proportion of repeats will occur purely by chance (i.e. where repeated motifs are not true repeats but just random sequence). While statistical models are available for predicting the extent to which this may occur [1], these do not help in determining which repeats can be classified as true repeats (i.e. those of structural/functional importance). There are no established methods for attempting to identify which repeats are functionally significant (that might reduce the need for robust experimental validation of each candidate). There are however, methods for looking at each repeat type (within the context of an individual proteome) that allow users to determine their own cut-off points (for instance, repeat length, or number of repeats). A closer look at the proteome of *L. infantum *shows that there are 974 proteins containing SAARs which are 6 residues or longer. If the minimum SAAR length is raised to 10 residues, then the number of repeat-containing proteins falls to 60 – a significant reduction. Table 4 shows the relationship between SAAR length and number of repeat-containing proteins as well as the proportion of DPRs and SRRs encountered when varying the number of those repeats searched for.

**Table 4:** Amino acid repeat distribution of selected species.

	**Single Amino Acid Repeats (SAARs)**									
	
	6+	7+	8+	9+	**10+**	11+	12+	13+	14+	15+
***L.infantum***	974	447	207	108	**60**	37	19	9	4	4
***T.brucei***	558	291	191	126	**86**	53	38	29	26	21
***D.discoideum***	6050	5361	4719	4197	**3741**	3374	3128	2920	2737	2585
***P.falciparum***	1904	1594	1346	1053	**853**	658	512	429	364	310

										
	**Di-peptide repeats (DPRs)**			**SRR repeats**						
	
	3+	**4+**	5+	2+	**3+**	4+				

***L.infantum***	507	**60**	25	531	**158**	108				
***T.brucei***	419	**97**	31	328	**177**	133				
***D.discoideum***	2318	**1003**	553	3676	**2060**	1122				
***P.falciparum***	745	**256**	119	1874	**1490**	1171				

Altering the search criteria can dramatically alter the number of proteins identified as bearing significant amino acid repeats. The values in bold indicate the chosen cut-off point for the analyses presented in this study.

While comparing repeat size against the number of proteins identified is a good method for identifying SAARs and DPRs, a different approach is required for determining the cut-offs for SRRs. Consider three proteins with different sequence lengths (100, 1000 and 10000 residues). A 10-residue motif repeated once is probably significant in the small protein, yet could have arisen by chance in the two larger proteins. This would suggest that when identifying real SRRs, the percentage of the protein which consists of the repeat region should be used. By contrast the same motif repeated 10 times in the largest protein would account for only 1% of the whole protein, yet could be structurally or functionally important. From this simple example, it is clear that defining a significant SRR requires a consideration of both repeat number and repeat size. Closer examination of the proteomes found in RepSeq suggests that sequences repeated at least three times typically account for large proportions (> 5%) of the whole protein (data not shown). For sequences repeated twice, only those which exceed 2–10% of the whole protein can be classified as non-random. Randomly occurring repeats typically account for < 1% of the total protein.

As discussed above, there is considerable flexibility in determining which cut-off values should be used when identifying repeat-containing proteins. For the purpose of the analyses presented here, the cut-offs established were SAARs of 10 residues or longer, DPRs of four repeats or more and SRRs repeated at least 3 times, using the normal repeat strength threshold setting. Analysis of the *Leishmania *and *Trypanosoma *proteomes found that repeat-containing proteins typically account for about 3–4% of the total protein number (Table 5). Interestingly, there is a large variation in the proportion of repeat-containing proteins in the *Plasmodium spp*. Those species that are pathogens of humans (*P.falciparum *and *P.vivax*) contain large numbers of repeat-containing proteins within their proteomes (33.49% and 21.62% respectively), while the rodent malaria species (*P.chabaudi*, *P.berghei *and *P.yoelii*) possess relatively few proteins of this type (2.19%, 4.05% and 13.49% respectively). There is also a large difference in the proteome sizes of *Plasmodium *species maintained in different hosts: the human infective species contain half the total number of predicted proteins as compared to the rodent pathogen species (although these numbers may change as the data sets undergo further annotation and refinement). It has previously been noted that *P. falciparum *contains a large number of low complexity repeat regions (predominantly coding for asparagines residues) due to single and multi-codon insertions within the coding region of the corresponding genes [19,20]. These repeat regions are believed to form non-globular segments of unknown function that extend from protein domains [23-26]. Further functional analysis is required to confirm these predictions. Interestingly, such low complexity repeat regions do not appear in the *P.vivax *and *P.yoelii *proteomes.

**Table 5:** Amino acid repeat frequency in protozoan parasitic proteomes.

**Species**	**Total Predicted Coding Sequences**	**SAARs (10+)**	**DPRs (4+)**	**SRR (3+ Repeats)**	Total amino acid repeat containing proteins *	**Total % repeat containing proteins**
***L.braziliensis***	7046	34	40	123	190	2.70%
***L.infantum***	8183	60	60	158	259	3.17%
***L.major***	8302	80	85	174	315	3.79%
						
***T.brucei***	8758	86	97	177	346	3.95%
***T.congolense***	17203	105	60	504	643	3.73%
***T.cruzi***	25401	594	245	514	1264	4.98%
						
***D.discoideum***	13498	3741	1003	2060	4627	34.28%
						
***E.histolytica***	9766	10	7	257	272	2.79%
						
***P.berghei***	12235	58	104	346	496	4.05%
***P.chabaudi***	15007	37	45	249	328	2.19%
***P.falciparum***	5479	853	256	1490	1835	33.49%
***P.vivax***	5352	111	113	1050	1157	21.62%
***P.yoelii***	8761	103	155	1024	1182	13.49%

* Some proteins contain several individual repeats. These are taken into account here.

The other species analysed in this study were the parasitic amoeba, *Entamoeba histolytica*, in which only 2.79% of the proteome can be classed as repeat-containing, and the soil amoeba, *Dictyostelium discoideum*, which contains the largest proportion of repeat-containing proteins (34.28%) of any of the protozoan proteomes analysed in this study. While SRRs typically account for the largest proportion of repeat-containing proteins in the species analysed, there are two notable exceptions: *T.cruzi *and *D.discodeum*, which both contain a larger proportion of SAARs. In the case of *T.cruzi*, this may be due to the large number of tyrosine, glutamine and glutamate SAARs that appear throughout the proteome. *D.discodeum*, like *P.falciparum*, contains very large numbers of low complexity repeat regions featuring asparagines.

RepSeq has primarily been designed with experimental parasitologists in mind. The ability to rapidly identify repeat-containing proteins (according to whatever criteria are set during the study) allows users to quickly generate lists of proteins for expression-profiling and functional analysis. An example of this is in the comparative proteomic analyses of different *Leishmania *species that cause diverse disease phenotypes [27]. In this project, the data provided by RepSeq has been further analysed to show that ~70% of repeat-containing proteins are conserved amongst all three species analysed. Furthermore, in nearly all cases, the repeat regions are particularly well conserved during speciation. A small number of the repeat-containing proteins are species-specific. Some of these are already targets for *Leishmania *researchers attempting to define virulence and pathogenicity factors, while others could provide interesting candidates for vaccine development.

## Conclusion

RepSeq provides an essential tool for the study of amino acid repeat-containing proteins. RepSeq compares favourably with other databases such as COPASAAR [1] and ProtRepeatsDB [19] due to its ability to quickly read through proteomes and present a comprehensive analysis which can be tailored to a wide variety of studies. Particular advantages are the ability to differentiate between the different repeat types and the ability to search for both very strict and very weak repeats. Furthermore, the sliding window employed by the algorithm is capable of identifying both perfect and mismatch repeats as long as a small part of the repeat is well conserved. While primarily designed for analysing lower eukaryotic organisms, RepSeq is capable of analysing proteomes from all species. The identification of amino acid repeat-containing proteins provides scientists with a new and complete subset of proteins which can be used in a range of studies from expression profiling to functional characterisation.

This may be of particular importance when studying pathogenicity and virulence factors in protozoan parasites [28] and also has applications to the study of neurodegenerative disease such as Huntington's chorea.

## Availability and requirements

RepSeq is freely accessible on the Internet at . The web-interface comprises many integrated sections for easy browsing and data retrieval and is supported with PERL and PHP scripts which enable formulation of queries against the database. All results are displayed either in tabulated or graphical forms.

## Authors' contributions

RPJL and DPD implemented the RepSeq algorithm and created the RepSeq website, RPJL designed and implemented the RepSeq database. DPD and DFS conceived the study, participated in its design and coordination and drafted the manuscript. All authors read and approved the final manuscript.
